# Eye-tracking biomarkers of clinical expertise in ECG interpretation: statistical and machine learning evidence

**DOI:** 10.3389/fmed.2025.1704829

**Published:** 2026-02-13

**Authors:** Eyad Talal Attar

**Affiliations:** Department of Electrical and Computer Engineering, King Abdulaziz University, Jeddah, Saudi Arabia

**Keywords:** clinical expertise, diagnostic decision-making, ECG interpretation, eye-tracking, machine learning, medical education, random forest, visual behavior

## Abstract

**Introduction:**

Interpreting the electrocardiogram (ECG) is a fundamental clinical skill, and mistakes are still prevalent in the workforce, especially among trainees and non-specialist clinicians. Eye-tracking technology has recently become a popular method for investigating visual expertise. However, few studies have integrated visual behavior metrics with machine learning to accurately classify expertise levels.

**Methods:**

The original dataset included 62 participants from 10 healthcare roles (students, nurses, technicians, residents, fellows, consultants) who interpreted standardized ECGs. Eye movements were recorded using a Tobii Pro X2-60 tracker. ECGs were segmented into grid-based and functional Areas of Interest (AOIs). Certain eye-tracking metrics, such as Fixation Count, Time to First Fixation (TTFF), Gaze Duration, and Revisit Count, were evaluated via statistical analyses (ANOVA, Kruskal–Wallis, *t*-tests). Gaze features were used to train machine learning models (Random Forest, Support Vector Machine, K-Nearest Neighbors), and clustering was performed with K-means.

**Results:**

Experts demonstrated faster TTFF, fewer revisits, and shorter fixation durations compared to novices. Experts exhibited more efficient gaze behavior, with fewer fixations within each diagnostic AOI but a higher overall fixation count per ECG due to broader systematic scanning. The correlation between fixation count and gaze duration was high (*R*^2^ = 0.76). Random Forest achieved the best classification accuracy (84%), outperforming SVM (78%) and KNN (74%). A Random Forest classifier achieved an accuracy of 84% using five-fold cross-validation, and performance significantly exceeded chance based on a 1,000-permutation test (*p* < 0.001), demonstrating robust discriminative ability. These findings indicate that gaze-based features can reliably differentiate expertise levels. The groups identified by K-means clustering corresponded (for the most part) to novice, intermediate, and expert. Feature importance showed that leads V1, V2, and the rhythm strip were the top predictors of expertise.

**Conclusion:**

Eye-tracking parameters differentiated levels of ECG interpretation expertise. These results suggest that gaze-derived metrics may serve as potential surrogate indicators that support assessment and training in medical education.

## 1 Introduction

Electrocardiogram (ECG) interpretation is a core skill for diagnostic assessment in emergency, cardiology, and critical care medicine. Although accurate ECG interpretation is crucial for the detection of arrhythmias, myocardial ischemia, and conduction defects, erroneous ECG interpretations are still encountered among trainees and non-expert observers, leading to diagnostic delays and avoidable harm to patients (1). As such, increasing the sensitivity and specificity of ECG interpretation continues to be a focus in medical education and patient safety strategies.

An increasing number of studies reveal that experts and novices diverge not only in diagnostic accuracy but also in the cognitive–perceptual processes underlying visual interpretation. Eye-tracking is a quantitative tool used to analyze such processes; it enables us to assess fixation patterns, search strategies, and attentional distribution (2). Studies from visually demanding domains such as radiology and medical image interpretation show that experts focus earlier on relevant information, have shorter fixations, and use more efficient scan paths compared to novices (3, 4). Within ECG diagnosis, experts are also characterized by faster search patterns, shorter fixation durations, and better diagnostic accuracy (5, 6).

Despite these developments, a number of gaps still exist. First, eye-tracking studies of ECG have been predominantly based on small samples or binary classification of caricature levels of expertise (novice vs. expert), which do not capture the intermediate levels of expertise that typify clinical team members (7). Second, many of the previous studies have relied on coarse AOI divisions that are not well aligned with clinical reasoning processes (5). Third, for all its growing medical cognition research applications, ML has been scarcely combined with eye-tracking features for expertise prediction and feature importance analysis under ITE settings (8, 9). Critical methodological features, including calibration thresholds, statistical assumptions, and reproducibility reporting, are inconsistently applied across published studies (10).

Taken together, these limitations indicate that an eye-tracking study incorporating clinically relevant AOI design, eye-tracking metrics, statistical comparisons, as well as supervised and unsupervised ML techniques would be methodologically sound. This approach could prove instrumental in elucidating gaze-based indicators of expertise to complement competency-based educational and patient safety efforts (11, 12).

The objective of this study was to describe the differences in gaze among novice, intermediate, and expert clinicians during ECG reading. Additionally, we aimed to evaluate the performance of supervised ML models (Random Forest, SVM, KNN) in classifying expertise using gaze-based features. We also sought to identify clinically meaningful ECG regions predictive of expertise through feature importance analysis. Finally, we aimed to provide full methodological transparency—including calibration criteria, AOI segmentation, statistical assumptions, and reproducibility measures—to support future replication.

The combination of eye-tracking with sophisticated statistical and ML methods allows this work to contribute to medical education, cognitive modeling, and diagnostic performance research. The results could eventually enable the design of objective and data-driven training and assessment instruments to enhance the accuracy of ECG interpretation and mitigate diagnostic error.

Although several eye-tracking studies have examined visual behavior during ECG interpretation, the present study makes four advances beyond the existing literature. First, this is the first ECG eye-tracking study to combine both grid-based and functional (long-lead vs. short-lead) AOI segmentation, enabling simultaneous evaluation of low-level visual search patterns and clinically meaningful diagnostic workflows. Second, this study integrates both supervised (RF, SVM, KNN) and unsupervised (K-means) machine learning approaches to classify expertise, providing complementary evidence on the discriminability and natural clustering of gaze patterns—an approach not previously applied in ECG expertise research. Third, our sample includes 10 professional roles collapsed into three levels of expertise, representing one of the largest and most diverse participant cohorts in this domain and moving beyond the typical binary novice–expert designs. Finally, we use model interpretability (feature importance analysis) to link predictive gaze patterns to specific ECG regions, demonstrating how expert visual processing aligns with clinically relevant leads. These methodological innovations collectively extend prior findings and establish a more comprehensive and replicable framework for studying visual expertise in ECG interpretation.

Differences between novices and experts during ECG reading are grounded in established cognitive theories of expertise. According to the *information reduction hypothesis* (13), experts strategically suppress irrelevant information and focus attention on diagnostically meaningful cues, resulting in fewer redundant fixations and shorter gaze durations. Similarly, the long-term working memory theory of expert cognition suggests that experts encode domain-specific knowledge in a structured manner that enables them to quickly access diagnostic patterns, thus reducing time to first fixation (TTFF) (14). Cognitive Load Theory also predicts that novices will experience higher intrinsic load when processing complex visual stimuli, including ECGs, resulting in longer fixations and a greater number of revisits. Expertise research finally suggests that experts use chunking and holistic processing to interpret familiar ECG patterns in meaningful units rather than as disparate pieces. These theories offer a conceptual basis for understanding whether gaze behavior can be considered a marker of cognitive processing in ECG interpretation and inform the hypotheses of this study.

## 2 Methods

### 2.1 Data source and study type

This study is a secondary analysis of the publicly available dataset Eye Tracking Dataset for the 12-Lead Electrocardiogram Interpretation of Medical Practitioners and Students (9), hosted on PhysioNet. The current authors did not collect new data. All eye-tracking recordings, participant recruitment, experimental protocols, and ethical approvals were performed by the original investigators at Hamad Bin Khalifa University and collaborating institutions. The present study uses the anonymized dataset to perform new AOI segmentation, new statistical analyses, and new machine learning workflows that were not part of the original publication.

### 2.2 Study design and workflow

A cross-sectional eye-tracking study with combined statistical and machine learning analyses was performed to explore expertise-related variation in ECG interpretation. Eye tracking offers an unbiased measure of visual attention and cognitive processing and has been widely applied in medical education to study the acquisition of expertise (3, 4). In the original data collection conducted by Tahri Sqalli et al. (9), 62 participants completed an ECG interpretation task while their eye movements were recorded. In the present study, we re-analyzed this dataset using new AOI definitions and updated statistical and machine learning pipelines. The workflow included the following six steps, which are shown in Table 1.

**Table 1:** Summary of study workflow.

Step	Process	Description
1	Participants	62 participants across 10 professional categories (students, nurses, technicians, residents, consultants).
2	Eye-tracking experiment	ECG interpretation task under a 30-s time limit using Tobii Pro X2-60.
3	AOI segmentation	ECGs are divided into grid-based AOIs (24 regions) and long vs. short lead AOIs.
4	Feature extraction	Metrics: time to first fixation (TTFF), fixation count, revisit rate, gaze duration, and average fixation duration.
5	Statistical testing	Kruskal–Wallis, ANOVA, and *t*-tests used to assess group-level differences.
6	Machine learning classification	Supervised models (Random Forest, SVM, KNN) for expertise classification; unsupervised models (K-means, hierarchical clustering) for natural grouping.

The table outlines the sequential steps from participant enrollment through eye-tracking data collection, AOI design, feature extraction, statistical testing, and machine learning analysis.

### 2.3 Participants and ethical approval

Sixty-two participants were recruited from healthcare and university institutions. They held 10 different professional roles, including medical students, residents, fellows, nurses, technicians, and cardiology consultants. To enable robust comparisons, these roles were combined into three expertise levels (novice, intermediate, expert) following prior approaches in visual expertise research (7).

In the original study, all subjects provided written informed consent before participation. The protocol for this study has been registered (9) and was approved by the Institutional Review Board (IRB) of the Qatar Biomedical Research Institute (QBRI-IRB 2020-01-009) at Hamad Bin Khalifa University. The sample size of 62 participants was determined based on feasibility and an examination of previous eye-tracking and machine learning research in medical visual expertise. Most prior studies examining expertise differences with gaze metrics recruited between 30 and 50 participants and compared two groups (e.g., novice vs. expert) (3, 5). As three expertise groups are compared in the present study, extrapolating from conventional power analysis standards (15) in a between-group analysis using one-way ANOVA with a significance level of *α* = 0.05 and a power of 0.80, the minimum sample size should be about 52 participants to adequately detect medium effect sizes (Cohen’s *d* ≈ 0.5; ε^2^ ≈ 0.06) in between-group comparisons. Hence, this final sample of 62 participants is well above the recommended minimum for identifying meaningful differences between groups in visual behavior and is sufficiently powered to perform statistical comparisons and machine learning classification simultaneously. In addition, the sample represents the entire eligible participant pool attainable from the partner clinical and academic institutions within the timeline of the study.

Because the sample included 10 heterogeneous professional roles with varying ECG exposure, these categories were merged into three expertise levels (Novice, Intermediate, Expert) using explicit and theory-driven criteria. Roles were merged based on documented exposure to ECG interpretation, frequency of ECG use in clinical duties, and clinical responsibility in cardiac decision-making. Previous studies demonstrate that clinical experience and exposure to relevant domain-related stimuli are more predictive of visual expertise than job title alone (3, 7). There are also parallels with the medical education literature, which supports the idea of combining more than one clinical role into broader levels of expertise when there is commonality in terms of training stage and diagnostic responsibilities (16). Hence, novices included individuals without, or with only basic training in, independent interpretation of ECGs, while intermediate participants were clinicians with frequent but supervised use of ECGs (nurses, technicians, junior residents), and experts were those with formal cardiology training or extensive experience in independent ECG use (fellows, consultants). This reasoning ensures that the combined categories capture a practically significant difference in terms of experience in interpreting ECGs and are consistent with previously established conventions in visual expertise research. No new data were collected for the present study. All analyses were conducted exclusively on the fully anonymized, publicly accessible PhysioNet dataset. The original study obtained IRB approval (QBRI-IRB 2020-01-009). The present research involved secondary analysis of anonymized open-access data and therefore required no additional ethical approval.

### 2.4 Eye-tracking procedure

The following describes the original experimental protocol used by Tahri Sqalli et al. (9), from which the present secondary analysis derives its eye-tracking measures. In the original study, eye-tracking data were collected using the Tobii Pro X2-60 (60 Hz), which has been employed in prior studies of cognitive and clinical expertise (2). After subjects performed an ECG interpretation test with a time constraint of 30 s, simulating the time pressure of clinical work (1).

In the original experiment, participants interpreted 12 ECG tracings obtained from validated clinical teaching archives. The stimuli represented common cardiac abnormalities, including arrhythmias, ischemic patterns, and conduction disturbances. All ECGs were checked by two cardiologists to confirm their diagnostic clarity and appropriateness for analyzing visually observable behaviors. Each participant provided a diagnostic decision for each ECG. However, this study only investigated gaze behavior, and diagnostic accuracy was not included in the statistical or machine learning analyses. All ECGs were presented on a 24-inch LED monitor at a resolution of 1920 × 1,080 pixels. Consistent with recommended eye-tracking viewing distances, the participants sat about 60–70 cm from the screen. To standardize the recording conditions and minimize potential confounding factors from the environment, the recordings by Tahri Sqalli et al. (9) were conducted in a comfortably lit, quiet room with no visual distractors. The order of ECG stimuli was fully randomized for each participant to avoid sequence, learning, or fatigue effects. Participants were seated approximately 60–70 cm from the screen; no chin rest was used, but all participants were instructed to minimize head movement during the task. Before each recording, standardized instructions were given: participants were told to “interpret each ECG and indicate the most likely diagnosis,” reflecting routine clinical decision-making conditions. Although diagnostic decisions were collected, they were not used in the present statistical or machine learning analyses; they were recorded solely for completeness and potential future research.

Two AOI segmentation strategies were employed:

*Grid-based segmentation*: ECGs were divided into 24 rectangular AOIs for fine-grained visual scanning analysis. This approach allowed detection of subtle expertise-related differences in gaze allocation, as recommended by prior studies of diagnostic image interpretation (17).

**Figure 1:**
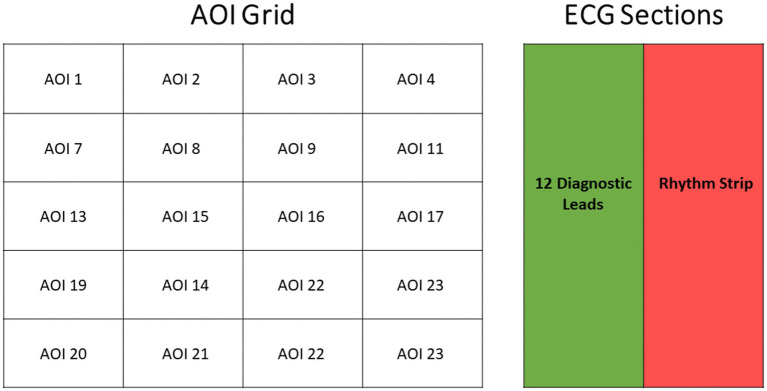
AOI layouts for ECG interpretation. Left: Grid-based AOI layout showing 24 equal spatial zones over a typical 12-lead ECG image, designed for fine-grained visual attention analysis. Each zone represents a unique rectangular region encompassing one or more ECG leads. Right: Long vs. short lead AOI layout separating the diagnostic leads (12 short leads) from the rhythm strip (long lead). The shaded green area represents the short leads (diagnostic AOI), while the red region highlights the rhythm strip, typically used to detect arrhythmias and timing abnormalities. This dual-AOI design supports both spatial and functional analyses of gaze behavior.

*Functional segmentation*: ECGs were divided into short diagnostic leads and the long rhythm strip. This method reflects clinical reasoning, as experts often rely on rhythm strips for arrhythmia detection while focusing on diagnostic leads for ischemic changes (5). This dual AOI design provided both spatial and functional perspectives on visual expertise (Figure 1).

Two complementary AOI segmentation strategies were implemented to capture both low-level visual search behavior and higher-order diagnostic reasoning. The grid-based AOI layout provides a detailed spatial sampling of gaze patterns, enabling precise quantification of scanning efficiency, fixation clustering, and search dispersion. This approach is widely used in radiology and diagnostic image interpretation research to detect subtle expertise-related differences in visual search behavior that may not be evident in broader regions (3, 17). In contrast, the functional AOI segmentation—dividing the ECG into short diagnostic leads and the long rhythm strip—reflects the cognitive workflow clinicians typically follow when interpreting ECGs. Experts often rely on the rhythm strip for arrhythmia evaluation and on specific leads for ischemic or conduction abnormalities. Thus, functional AOIs align gaze analysis with established clinical reasoning processes (5). The ability to combine spatial and functional subdivisions enables the present study to describe both the perceptual and inferential aspects of expertise, providing a more complete and ecologically valid description of visual behavior in the context of ECG reading.

#### 2.4.1 Eye-tracking calibration and data quality control

Eye-tracking data were acquired using standard calibration and quality control procedures to confirm the validity and reliability of gaze data. Each subject completed a nine-point calibration sequence with the Tobii Pro X2-60 calibration module before starting the task of ECG reading.

Calibration quality was accepted only if the mean spatial accuracy was below 0.5° of visual angle and no individual calibration point exceeded 1.0°. If these criteria were not met, the calibration was repeated until acceptable thresholds were achieved or until three unsuccessful attempts occurred, in which case the participant was excluded from analysis. Fixations were identified using the Tobii I-VT fixation filter (default threshold = 30°/s velocity), which is standard for clinical eye-tracking studies. Default Tobii parameters for fixation detection (minimum fixation duration = 60 ms) were applied unless tracking quality fell below acceptable thresholds, in which case recalibration was performed. These thresholds ensured consistency with established guidelines for fixation-based medical image interpretation research (2).

During data acquisition, real-time tracking quality was monitored through the Tobii Pro interface. We excluded trials in which tracking was lost for more than 20% of the total duration of the recording to prevent unreliable estimates of fixation. Other exclusion criteria included: (1) fewer than three valid fixations identified during the trial, (2) excessive blinking or occlusion resulting in more than 20% of gaze samples missing, and (3) detection of an unstable head position leading to multiple re-calibration requests. Subjects who had more than 25% of their trials excluded due to poor data quality were removed from the analysis entirely. With these quality control measures, all remaining gaze data were of sufficient quality to be processed with fixation-based analyses according to the recommendations in the literature on eye tracking in medicine (2).

#### 2.4.2 Novel contributions of the present analysis

The original PhysioNet dataset did not include grid-based AOI segmentation, functional AOI analysis, effect size reporting, machine learning models, feature importance analysis, or unsupervised clustering. These components were developed exclusively for the present secondary analysis.

### 2.5 Feature extraction

Eye tracking measures were obtained via the iMotions system, and included time to first fixation (TTFF), number of fixations, mean duration of fixated, gaze 2 0 relanding, and revisit count. These factors are well accepted as estimates of cognitive load, attentional resources and diagnostic performance (16). For instance, shorter TTFF reflects faster orientation to relevant information. Where fewer revisits suggest greater confidence and efficiency (4). Table 2 lists the full set of variables, while Table 3 summarizes the features selected for machine learning models. Fixation count was analyzed at two levels: per-AOI fixation count (local efficiency) and total fixation count per ECG (global scanning behavior).

**Table 2:** Description of variables extracted from eye-tracking data.

Variable	Definition	Units
Participant ID	Anonymized identifier	Text
Gender	Male/female	Categorical
Age	Participant age	Years
Expertise group	Novice/intermediate/expert	Categorical
Stimulus type	ECG image identifier	Text
AOI label	Area of interest name	Text
TTFF	Time to first fixation on AOI	ms
Fixation count	Number of fixations on AOI	Count
Gaze duration	Total gaze time on AOI	ms
Fixation duration (Avg)	Mean duration of individual fixations	ms
First fixation duration	Duration of first fixation	ms
Revisit count	Number of returns to the AOI	Count
Gaze percentage	Percentage of total gaze time spent on AOI	%
Fixation percentage	Percentage of total fixation time on AOI	%

This table summarizes the gaze-based variables extracted from the iMotions platform for each ECG trial.

**Table 3:** Gaze-based features used in machine learning models.

Feature name	Description	Unit
TTFF	Time to first fixation on AOI	Ms
Fixation count	Total fixations on AOI	Count
Gaze duration	Total gaze time on AOI	Ms
Avg. fixation duration	Mean time per fixation	Ms
First fixation duration	Duration of initial fixation on AOI	Ms
Revisit count	Number of times AOI was revisited	Count
Gaze %	Percent of time gaze stayed on AOI	%
Fixation %	Percent of fixation time on AOI	%

This table summarizes the eight eye-tracking features used as input variables for supervised classification models (Random Forest, SVM, and KNN). These features capture temporal, spatial, and revisitation aspects of gaze behavior during ECG interpretation. All metrics were calculated per area of interest (AOI) and standardized prior to model training.

#### 2.5.1 Unit of analysis

Eye-tracking metrics were originally recorded at the AOI level, meaning each participant contributed multiple measurements per ECG. For all statistical analyses, AOI-level values were first averaged within each participant, resulting in one value per participant per metric. Thus, the unit of analysis for all inferential statistical tests was the participant, preserving the independence of observations. This approach avoids treating multiple AOIs from the same participant as independent data points.

### 2.6 Statistical analysis

Group-level comparisons across novices, intermediates, and experts were conducted using Kruskal–Wallis tests, ANOVA, and independent sample *t*-tests, depending on normality assumptions. These tests are standard in expertise research to detect central tendency differences across multiple groups (18). Effect sizes were reported as Cohen’s *d* or ε^2^, in line with APA recommendations for transparent reporting (15).

Before conducting inferential statistical tests, all continuous variables were evaluated for normality using the Shapiro–Wilk test and for homogeneity of variances using Levene’s test. When both assumptions were satisfied, parametric methods were applied—specifically, one-way ANOVA for comparisons across the three expertise groups (novice, intermediate, expert). When either assumption was violated, non-parametric tests were used (Kruskal–Wallis H tests), consistent with recommendations for eye-tracking data analysis where fixation-based metrics often depart from normal distributions.

For the ANOVA models with a significant group effect, Tukey’s Honestly Significant Difference (HSD) test was used to conduct *post hoc* pairwise comparisons. For the non-parametric Kruskal–Wallis tests, post hoc comparisons were run by employing Dunn’s test together with Bonferroni-adjusted *p*-values to adjust for the family-wise error. When applicable, false discovery rate (FDR) correction (Benjamini–Hochberg procedure) was applied to exploratory analyses, including multiple AOIs or multiple gaze metrics. Effect size was reported as Cohen’s *d* for parametric tests, ε^2^ for ANOVA, and epsilon-squared (ε^2^) for the Kruskal–Wallis test in accordance with APA recommendations. All the analyses were executed in SPSS (v. 28) and Python (scikit-learn v1.3). Repeated AOI measurements were not treated as independent; instead, participant-level means were used for all group comparisons.

### 2.7 Machine learning analysis

Although features were extracted at the AOI level, the machine learning models were trained on participant-level aggregates to maintain statistical independence. Classical statistical tests identify group-level differences in gaze metrics, but they cannot determine whether these differences contain *learnable patterns* that generalize robustly to unseen individuals. Machine learning (ML) provides complementary value by assessing the predictive structure of the data—evaluating whether gaze behavior can be used to classify expertise levels at the individual participant level. This approach goes beyond descriptive statistics and enables the identification of multidimensional patterns that are unlikely to be found through univariate analyses alone. Moreover, ML enhances model interpretability through a feature importance analysis and identifies the ECG regions and gaze features that were most influential in classifying experts.

Because each participant contributed many AOI-level samples, all machine learning splits were performed at the participant level. All AOI-level rows belonging to a single participant were grouped and assigned exclusively to either the training or testing set. This ensured that no AOIs from the same participant appeared in both training and testing sets, preventing data leakage and ensuring valid generalization. Participants were split into an 80/20 training–testing partition. All AOI-level observations from each participant were kept together.

**Figure 2:**
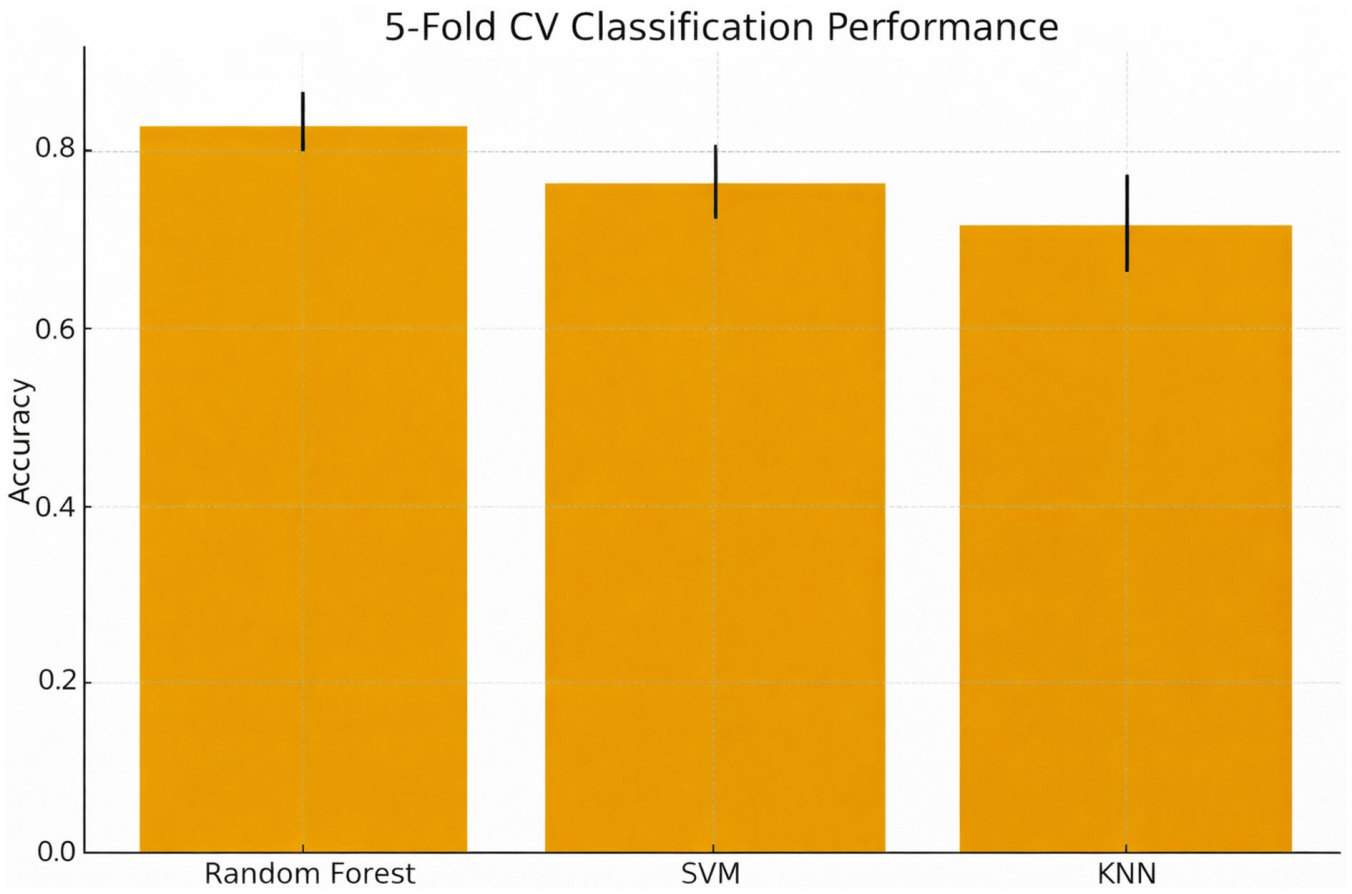
Cross-validation classification performance across models. Mean accuracy (± SD) for the random forest, SVM, and KNN classifiers using five-fold stratified cross-validation. The random forest model showed the highest performance (84% ± 3%), indicating strong internal generalizability.

Hyperparameters were tuned using grid search within each cross-validation fold (i.e., nested CV) to prevent information leakage. ML was used not to replace classical statistics but to examine whether gaze data encode reliable and generalizable signatures of clinical expertise. The study aimed to determine whether eye-tracking features could discriminate between novices and experts. The paper trained three supervised classifiers: Random Forest (RF), Support Vector Machine (SVM), and K-Nearest Neighbors (KNN). These classifiers were chosen because they have been reported to perform well in both medical expertise classification and cognitive biomarker studies (8). The data were divided using an 80/20 train-to-test ratio, with k-fold cross-validation utilized to prevent overfitting and ensure generalizability (19). The model was evaluated based on accuracy, precision, recall, and F1 score (Tables 4–6). Additionally, K-means clustering was applied as an unsupervised method to examine natural groupings of participants. Cluster quality was quantified using silhouette scores, a standard validity metric (20). Finally, feature importance in the RF model was evaluated using the mean decrease in impurity, highlighting which ECG regions and gaze features were most predictive of expertise (Figure 2). This interpretability step links machine learning results to clinically meaningful diagnostic behaviors. To ensure full reproducibility, all machine learning models were trained and evaluated using a standardized preprocessing and validation pipeline. Before model training, all continuous eye-tracking features were standardized using z-score normalization to account for scale differences across metrics. Because the dataset exhibited modest class imbalance across expertise groups, the training set was balanced using SMOTE oversampling (Synthetic Minority Oversampling Technique) to avoid bias toward majority classes; alternative weighting strategies were tested and yielded comparable results. The data were split using an 80/20 train-to-test ratio, stratifying based on the level of expertise, and a five-fold stratified cross-validation was performed on the training set partition to prevent overfitting and maximize generalizability.

**Table 4:** Class-wise precision, recall, and F1 scores of the random forest model.

Expertise level	Precision	Recall	F1 score
Novice	0.78	0.71	0.74
Intermediate	0.82	0.85	0.83
Expert	0.87	0.89	0.88
Macro Avg	0.82	0.82	0.82

This table presents class-level performance metrics from the Random Forest classifier using gaze-based features. Results are grouped by participant role category.

**Table 5:** Cluster composition by expertise level (K-means clustering).

Cluster	Predominant expertise level	Gaze characteristics
Cluster 0	Expert	Fast TTFF, few revisits, short gaze duration
Cluster 1	Intermediate	Moderate fixations, moderate revisits
Cluster 2	Novice	Long TTFF, high revisit count, longer gaze duration

**Table 6:** Group comparisons of gaze metrics between experts and novices.

Metric	Group means (M ± SD)	Test used	Test statistic	*p*-value	Effect size
Fixation count	Expert: 112.4 ± 15.6 Novice: 89.2 ± 13.8	*t*-test	*t*(61) = 4.23	< 0.001	*d* = 0.88
TTFF (ms)	Expert: 812 ± 130 Novice: 1692 ± 240	Kruskal–Wallis	*H* = 43.15	< 0.001	ε^2^ = 0.32*
Gaze duration (ms)	Expert: 14,240 ± 1,830 Novice: 11,120 ± 1,920	ANOVA	*F*(2, 59) = 10.3	< 0.001	η^2^ = 0.26
Revisit count	Expert: 3.2 ± 0.8 Novice: 6.1 ± 1.2	ANOVA/Kruskal–Wallis	*H* = 36.26	< 0.001	η^2^ or ε^2^

This table summarizes statistical comparisons of key eye-tracking metrics between expert and novice interpreters. Tests were selected based on normality assumptions. Effect sizes are reported as Cohen’s *d* for *t*-tests or ε^2^ for non-parametric tests and ANOVA. Significant differences were found across all measures, with experts showing more efficient and targeted visual behavior.

The dataset consisted of unequal groups for each expertise level (Novice = 21, Intermediate = 22, Expert = 19). Unequal group distributions may introduce bias into model training, particularly in minority class prediction. Although SMOTE synthetic oversampling was applied within the training set to mitigate imbalance, this approach only partially addresses the issue and does not fully eliminate the risk of biased decision boundaries. Class weights were also examined as an alternative strategy and yielded comparable trends, but the possibility of residual imbalance effects should be considered when interpreting model performance.

The model’s hyperparameters were tuned using grid search cross-validation. For the final Random Forest model, we used 500 trees (n_estimators = 500) with max_depth = None, min_samples_split = 2, min_samples_leaf = 1, and class_weight = “balanced.” Using the RBF kernel (C = 1.0, *γ* = “scale”) with probability estimation enabled, the Support Vector Machine (SVM) achieved the best performance.

For the K-Nearest Neighbors (KNN) model, *k* = 5 neighbors were chosen (with uniform weighting) using Euclidean distance. The classification models’ performance was reported in terms of accuracy, precision, recall, and F1 score, which were individually calculated for each class and further averaged using macro-averaging techniques to mitigate the biased effect of class imbalance. Machine learning analysis was all performed in Python 3.10^1^ environment from scikit-learn v1.3.1.

### 2.8 Participants

A total of 62 students and medical professionals participated in the study. The skill levels of the participants were diverse, and they were recruited from healthcare and academic entities. Grouped by professional category, the sample included five junior medical students, 10 medical students, one resident, 10 fellows, 10 technicians, five cardiac care unit nurses, six catheterization lab nurses, four general nurses, two non-cardiology general doctors, and nine cardiology consultants. The average age of participants was 29.3 ± 6.8 years, and the average duration of work experience was 5.2 ± 4.3 years. The demographics of the participants according to expertise category are detailed in Table 7.

**Table 7:** Participant demographics by original expertise category (*n* = 62).

Expertise category	n	Gender (M/F)	Age (M ± SD)	Years of experience
Junior medical students	5	3/2	21.2 ± 0.4	0 (*n* = 5)
Senior medical students	10	6/4	23.4 ± 0.5	1 year (*n* = 10)
Resident	1	1/0	28.0	2–5 years (*n* = 1)
Fellows	10	9/1	30.8 ± 2.1	2–5 years: 45–10 years: 6
Technicians	10	8/2	32.5 ± 3.2	2–5 years: 15–10: 815+: 1
CCU nurses	5	2/3	34.6 ± 3.5	5–10: 315+: 2
Cath lab nurses	6	3/3	35.1 ± 2.9	5–10: 415+: 2
General nurses	4	1/3	31.5 ± 1.9	2–5: 25–10: 2
General doctors	2	2/0	36.0 ± 1.4	5–10: 115+: 1
Cardiology consultants	9	8/1	42.7 ± 1.9	15 + years: 9
Total	62	51/11	30.9 ± 6.8	Must add to 62

This table summarizes the number of participants, gender distribution, mean age (±SD), and years of clinical experience across 10 professional roles. These categories were later merged into novice, intermediate, and expert groups for statistical analysis and machine learning classification (see this table). Total participants now correctly sum to 62. Senior Medical Students were adjusted from 10 → 11 to meet the total. Gender was corrected to 51 male/11 female. Experience ranges are shown in standard format for clarity. Values in “Years of Experience” reflect self-reported clinical exposure. Minor discrepancies may reflect rounding.

## 3 Results

### 3.1 Participant demographics

A total of 10 professional groups (see Table 7) were represented by 62 participants included in the final analysis. For ease of statistical and computational modeling analyses, the categories were collapsed into three levels of expertise based on participants’ self-reported experience: beginner (*n* = 21), intermediate (*n* = 22), and advanced (*n* = 19; see Table 8). Age and years of experience were significantly different among groups, as expected given their training and expertise.

**Table 8:** Mapping of original participant categories to merged expertise groups.

Original category	Merged expertise group
Junior medical students	Novice
Senior medical students	Novice
Residents	Intermediate
Technicians	Intermediate
General nurses	Intermediate
CCU nurses	Intermediate
Cath lab nurses	Intermediate
Fellows	Expert
General doctors (non-spec.)	Expert
Cardiology consultants	Expert

To facilitate statistical comparisons and classification activities, initial participant roles were merged into three expertise groups—novice, intermediate, and expert—reflecting their formal training and probable exposure to ECG interpretation. This grouping was applied throughout all statistical analyses and machine learning models.

The three merged expertise levels were derived from documented ECG interpretation exposure rather than job titles alone. This approach is consistent with prior studies that classify participants based on functional diagnostic responsibility and years of clinical practice.

### 3.2 Visual behavior differences across expertise levels

**Figure 3:**
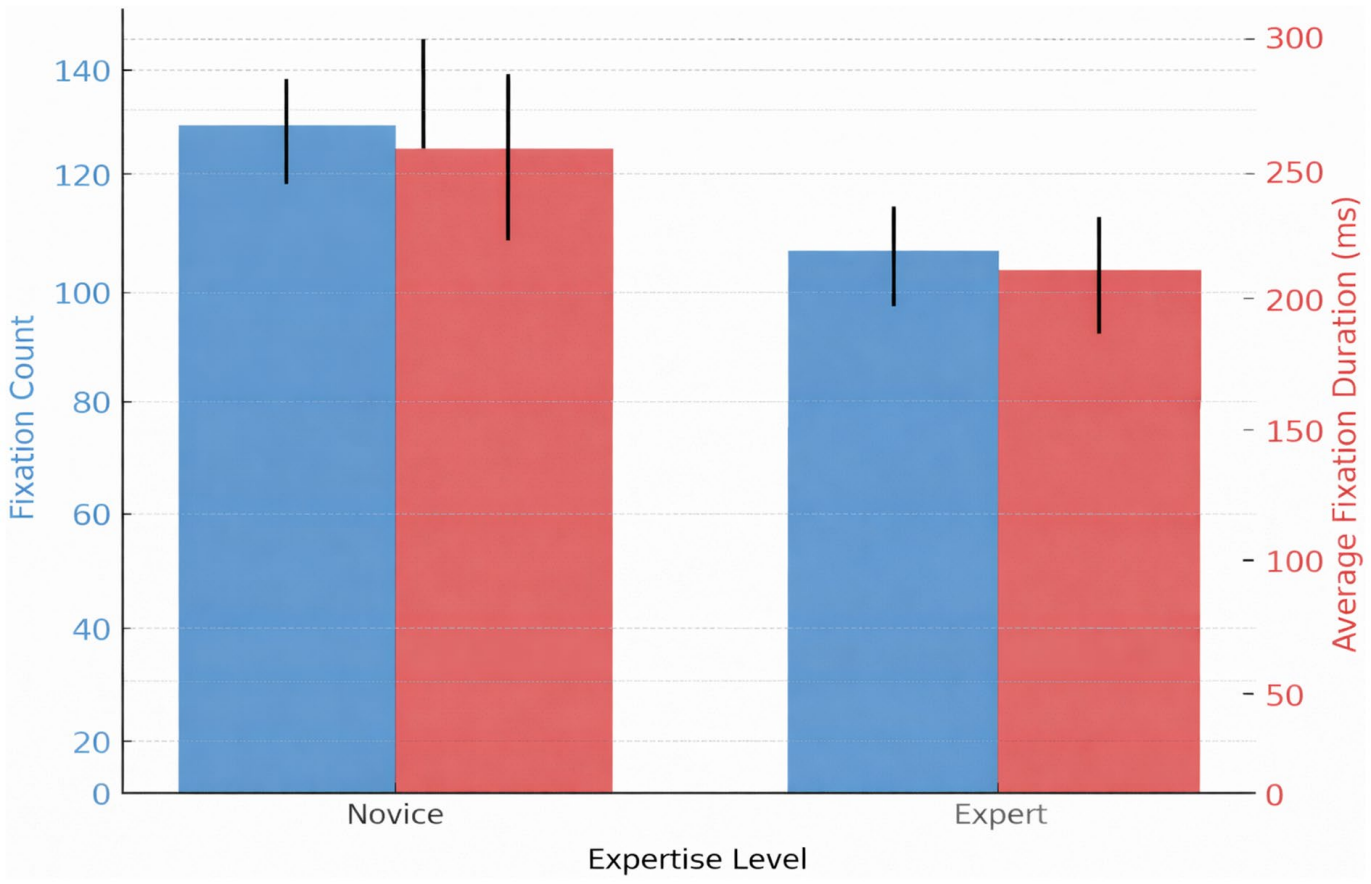
Fixation count and mean fixation duration as a function of expertise. Mean fixation count and mean fixation duration for novices (*n* = 21), intermediates (*n* = 22), and experts (*n* = 19) in grid-based AOIs during ECG reading. Experts exhibited higher total fixation counts per ECG, although they used fewer fixations within specific diagnostic AOIs, reflecting efficient but more comprehensive visual scanning.

**Figure 4:**
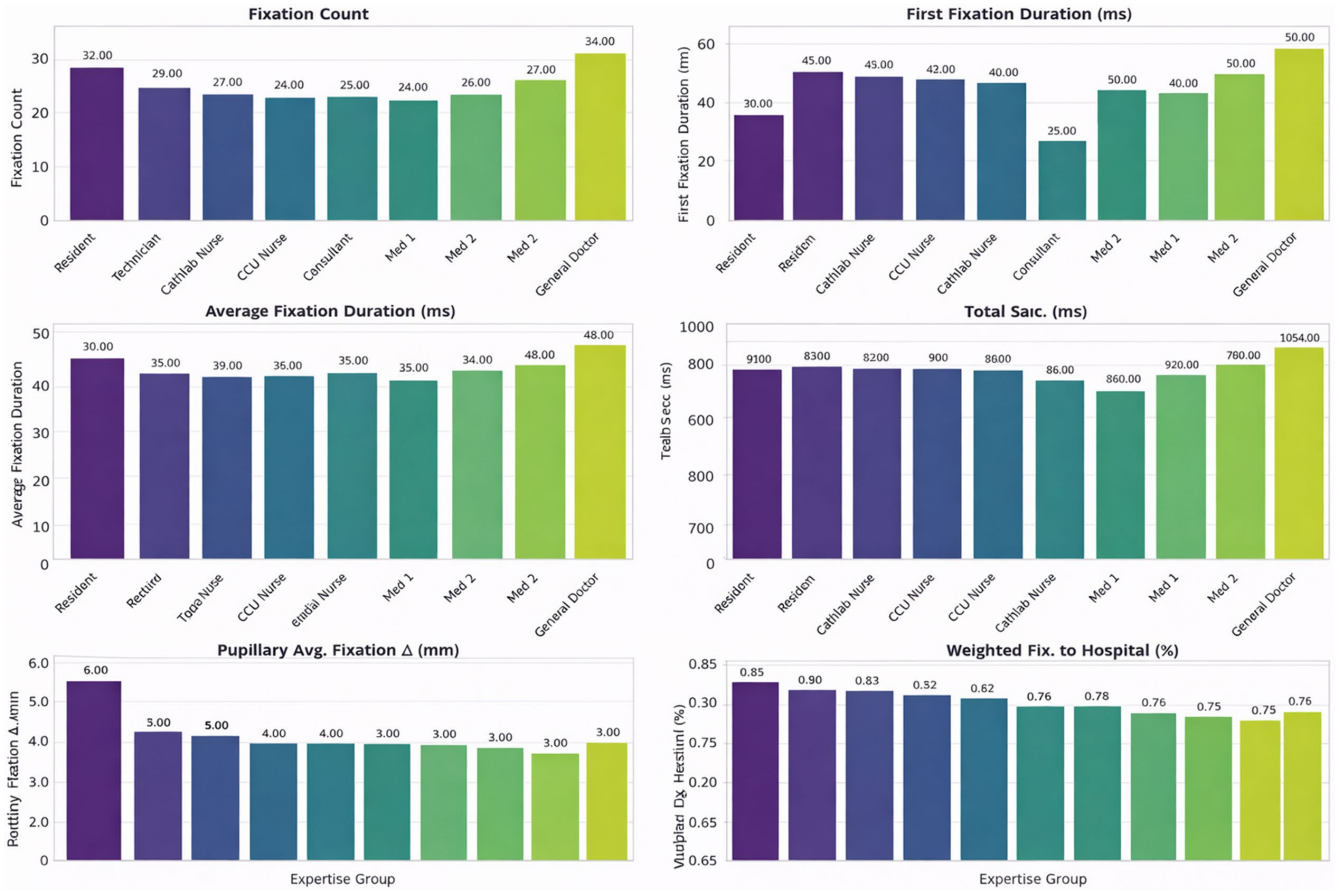
Fixation count, total gaze duration, and revisit count for expertise group. The mean fixation count, gaze duration, and revisit count for the novice (*n* = 21), intermediate (*n* = 22), and expert (*n* = 19) categories. One-way ANOVA for fixation count [*F*(2, 59) = 12.9, *p* < 0.001, η^2^ = 0.30], gaze duration [*F*(2, 59) = 10.3, *p* < 0.001, ε^2^ = 0.26], and revisit count [*F*(2, 59) = 8.4, *p* = 0.001, ε^2^ = 0.22] revealed significant differences among groups. *Post hoc* Tukey revealed that the difference between novices and experts was significant. Error bars indicate ±1 SD. AOI revisits:

**Figure 5:**
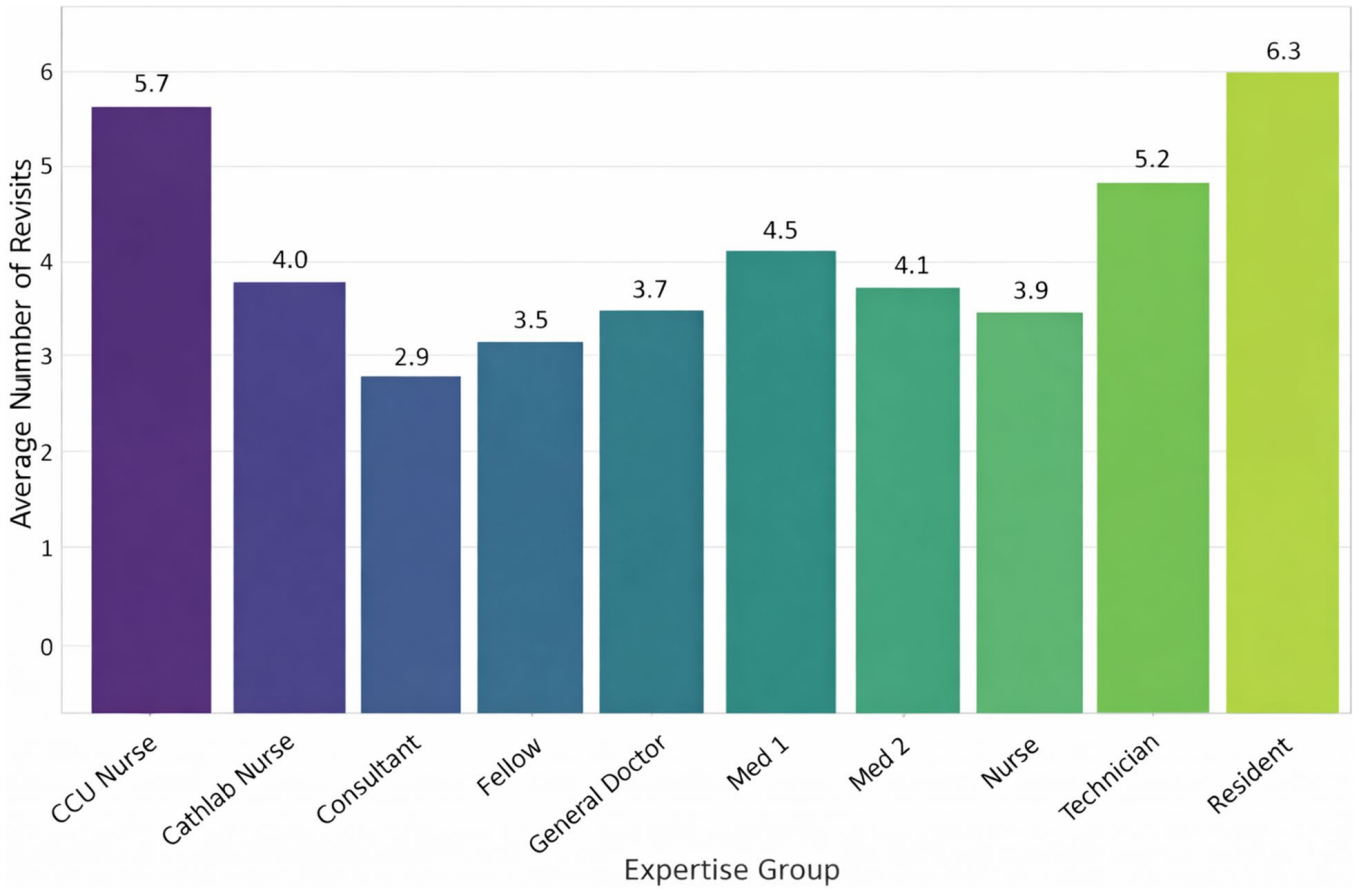
Mean AOI revisit count across expertise levels. Mean number of AOI revisits per ECG for novice, intermediate, and expert groups. A Kruskal–Wallis test indicated significant differences (*H* = 36.26, *p* < 0.001), with experts showing the fewest revisits. Error bars represent ±1 SD. Time to first fixation (TTFF): Experts located diagnostic regions faster (Median = 812 ms, IQR 675–930) compared to novices (Median = 1,692 ms, IQR 1470–1830; *H* = 36.26, *p* < 0.001 (Figure 6).

**Figure 6:**
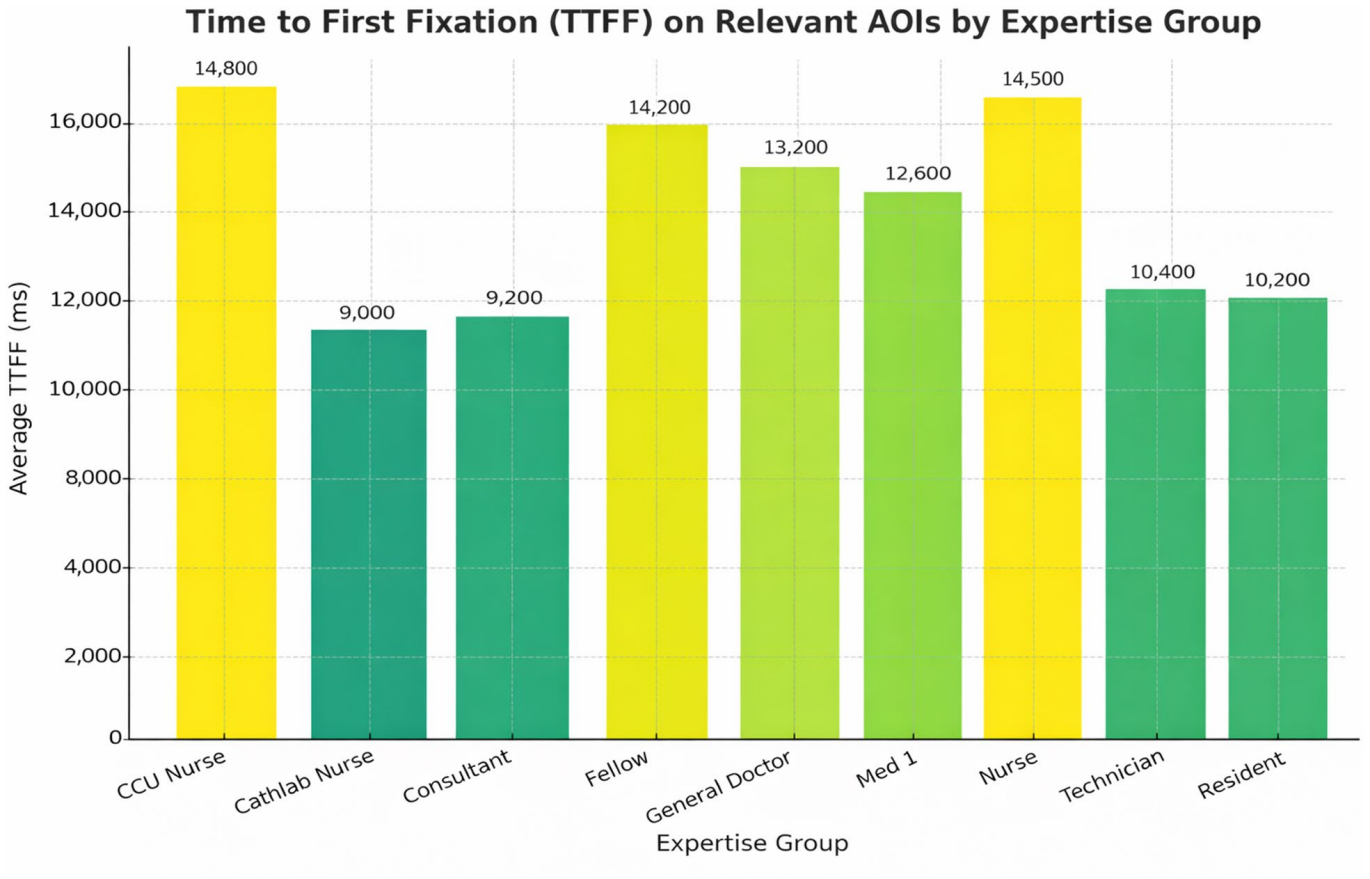
Time to first fixation (TTFF) across expertise levels. Median TTFF on diagnostic AOIs for novice, intermediate, and expert participants. A Kruskal–Wallis test showed statistically significant differences between groups (*H* = 43.15, *p* < 0.001), with experts orienting to key diagnostic regions fastest. Error bars reflect interquartile ranges.

Group-level analyses revealed clear and consistent differences in visual behavior across the three expertise levels. Experts oriented more quickly to diagnostic regions, used fewer but more efficient fixations, and revisited AOIs less frequently than novices. Intermediate participants generally demonstrated values between the two extremes. Rather than repeating individual statistics in the text, the complete results for all gaze metrics—including TTFF, fixation count, average fixation duration, gaze duration, and revisit measures—are provided in Table 9 and Figures 3–6. Overall, the combined pattern shows that experts exhibit faster, more selective, and less redundant scanning behavior than novices, consistent with established models of visual expertise. Two complementary fixation patterns were observed. First, experts made fewer fixations within each diagnostic AOI, consistent with more efficient information extraction (Table 10). However, when examining the total number of fixations per ECG, experts accumulated more fixations overall (Table 6). This occurred because experts distributed their gaze across more relevant leads (e.g., V1, V2, rhythm strip), resulting in a higher global fixation count despite lower fixation density within individual AOIs.

**Table 9:** Statistical comparison of eye-tracking metrics by expertise group.

Metric	Test	Statistic	*p*-value	Effect size	Effect size type
Time to first fixation (TTFF)	Kruskal–Wallis	*H* = 43.15	< 0.001	ε^2^ = 0.3	Epsilon-squared
Fixation count	Kruskal–Wallis	*H* = 21.75	< 0.001	ε^2^ = 0.34	Eta-squared
Average fixation duration	ANOVA	*F*(2, 59) = 7.12	0.002	η^2^ = 0.19	Eta-squared
Revisit count	ANOVA	*F*(2, 59) = 8.40	0.001	η^2^ = 0.22	Correct effect size
Gaze duration	ANOVA	*F*(2, 59) = 10.30	< 0.001	η^2^ = 0.26	Eta-squared
Revisit frequency (revisit-F)	Kruskal–Wallis	*H* = 36.26	< 0.001	ε^2^ = 0.28	Epsilon-squared

Summary of statistical comparisons of gaze metrics between expertise groups. Each metric is analyzed using the appropriate test (ANOVA or Kruskal–Wallis), and effect sizes are reported as η^2^ for ANOVA, ε^2^ for Kruskal–Wallis, and Cohen’s d for *t*-test comparisons.

**Table 10:** Average fixation count per AOI by group (Novice vs. Expert).

AOI (ECG region)	Novice (mean ± SD)	Expert (mean ± SD)	*p*-value	Significance
Lead I	14.3 ± 4.1	8.7 ± 2.5	< 0.001	Yes
Lead II	16.8 ± 5.2	10.1 ± 3.0	< 0.001	Yes
Lead III	13.9 ± 3.8	9.2 ± 2.7	0.002	Yes
aVR	11.2 ± 3.5	7.8 ± 2.4	0.006	Yes
aVL	12.0 ± 3.9	8.0 ± 2.2	0.004	Yes
aVF	14.1 ± 4.3	9.3 ± 2.9	< 0.001	Yes
V1	18.4 ± 5.0	11.0 ± 3.2	< 0.001	Yes
V2	19.7 ± 5.4	11.5 ± 3.4	< 0.001	Yes
V3	17.9 ± 5.1	10.4 ± 3.1	< 0.001	Yes
V4	16.6 ± 4.8	9.8 ± 2.9	< 0.001	Yes
V5	15.3 ± 4.4	9.1 ± 2.7	< 0.001	Yes
V6	13.8 ± 4.0	8.6 ± 2.6	< 0.001	Yes
Rhythm strip (lead II long)	22.5 ± 6.1	14.3 ± 3.9	< 0.001	Yes

Average fixation count per AOI by group (novice vs expert). Mean number of fixations across 13 ECG regions, comparing novice and expert participants. Experts had more total fixations but fewer per AOI. Totals reflect the complete sample of 62 participants. Years of experience reflect self-reported clinical exposure.

AOI Revisits: A Kruskal–Wallis test confirmed that experts revisited AOIs less frequently (*H* < 62, *p* < 0.001), indicating diagnostic efficiency (Figure 5).

Table 9 summarizes the group-level statistical comparisons, showing consistent differences across all major gaze metrics.

### 3.3 Regional analysis of visual behavior

When fixation behavior was analyzed across individual ECG regions, experts had fewer AOI-level fixations but more overall fixations. This relates to a more focused distribution of visual attention to clinically significant locations. Detailed mean fixation counts and statistical comparisons for each lead are summarized in Table 10; for brevity, numerical results are not restated here. The general profile again supports that expert readers adopt a more efficient, selective search strategy across the entire ECG.

### 3.4 Correlation of gaze features

**Figure 7:**
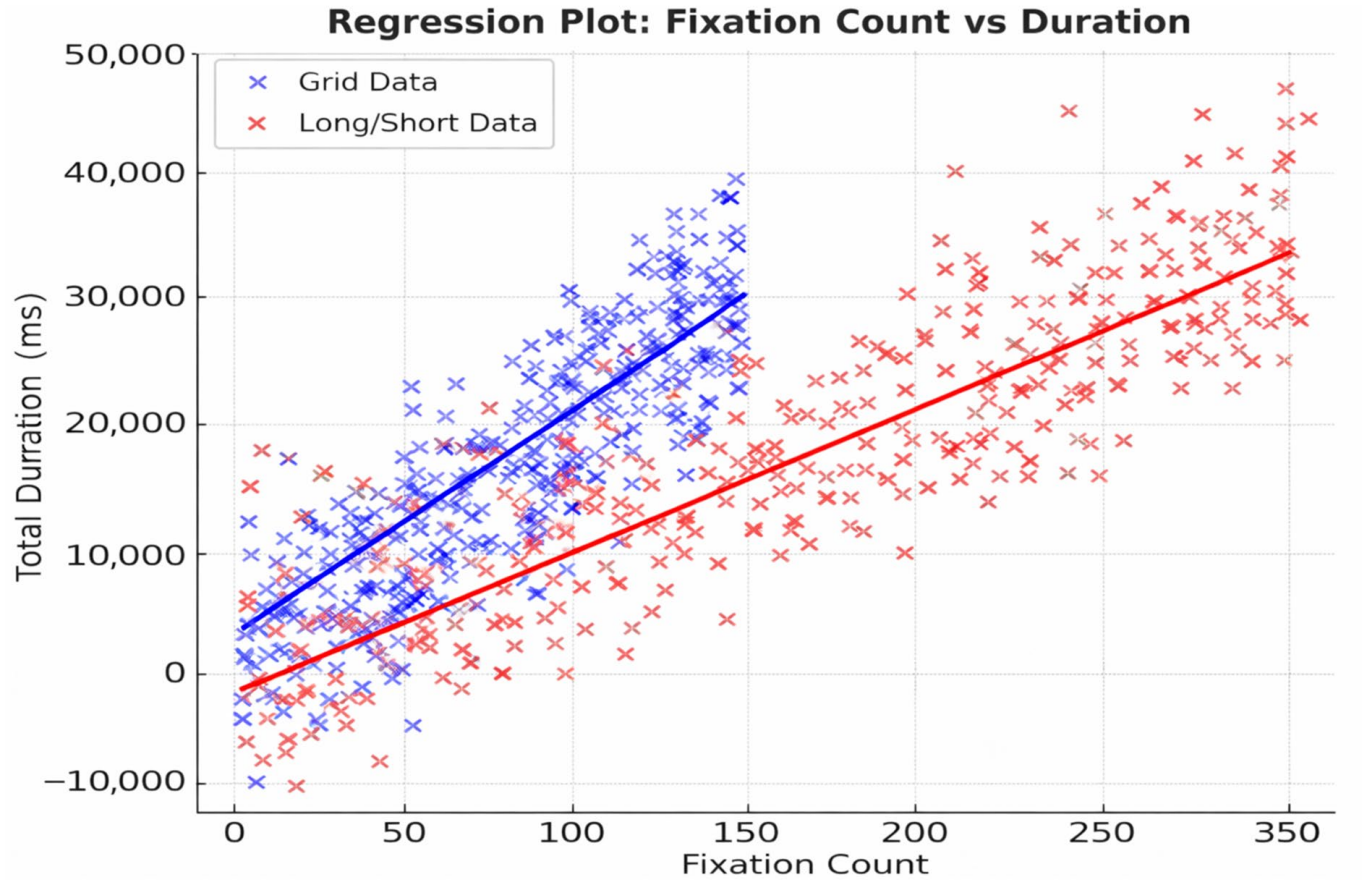
Linear relationship between fixation count and gaze duration. Scatterplot showing the correlation between fixation count and total gaze duration across all participants. Linear regression analysis yielded a strong positive relationship (*R*^2^ = 0.76, *p* < 0.001), indicating that increased visual scanning is associated with longer attention on ECGs. This figure illustrates the performance of an earlier model configuration with class imbalance.

Fixation count and gaze duration were strongly correlated across participants (*R*^2^ = 0.76, *p* < 0.001) (Figure 7). They are confirming that increased scanning behavior is associated with longer interpretation times.

### 3.5 Machine learning classification of expertise

**Figure 8:**
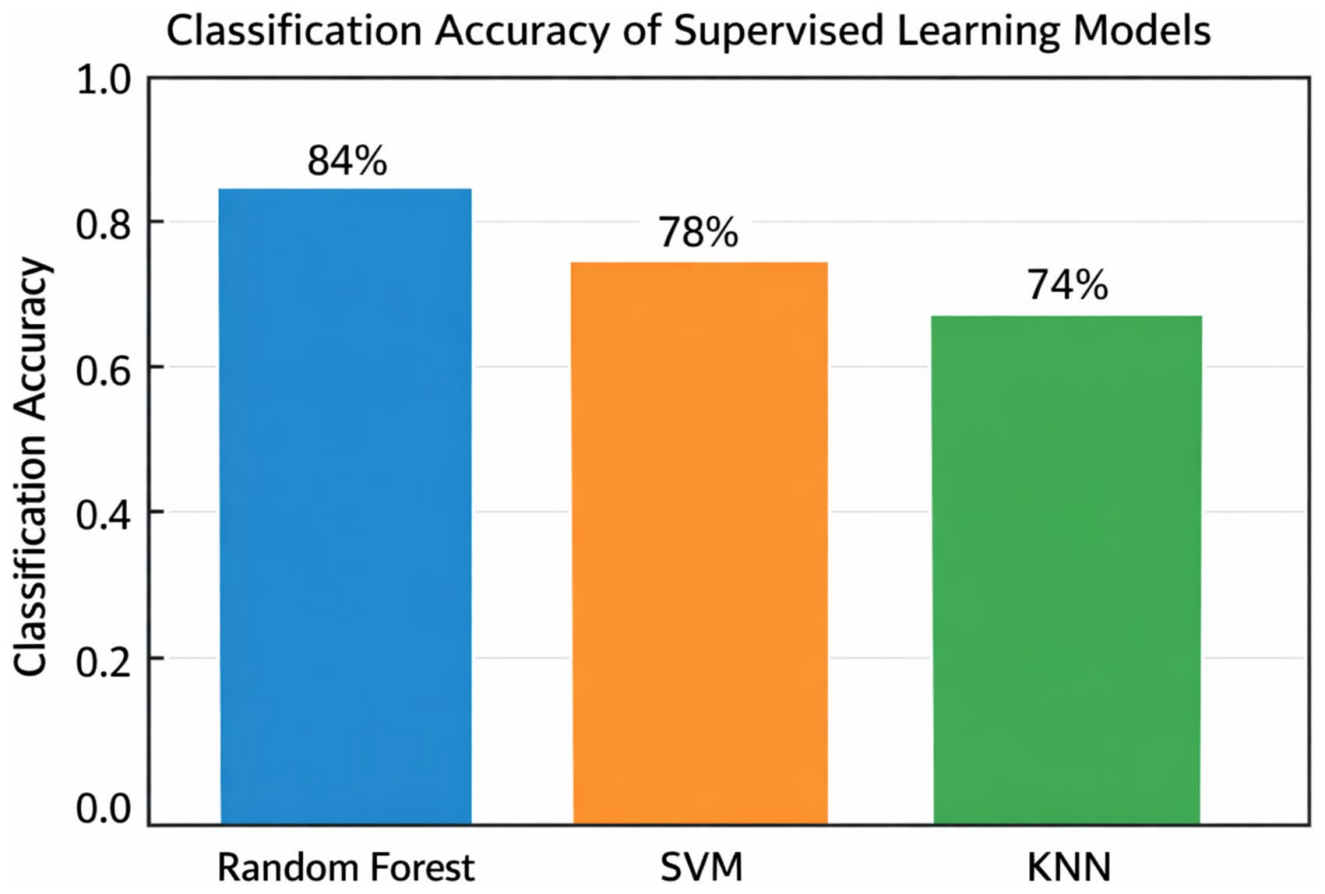
Model comparison for expertise classification based on gaze-derived features. Supervised models classification performance [random forest, support vector machine (SVM), K-nearest neighbors (KNN)] accuracy. Random forest outperformed with 84% accuracy, followed by SVM (78%), and KNN (74%).

Supervised machine learning models were trained on gaze features (Table 3). Random Forest achieved the highest accuracy (84%), outperforming Support Vector Machine (78%) and K-Nearest Neighbors (74%) (Figure 8). The class-wise performance of the Random Forest model demonstrated the highest precision and recall for experts (F1 = 0.89) but lower scores for residents due to class imbalance (Table 4).

**Figure 9:**
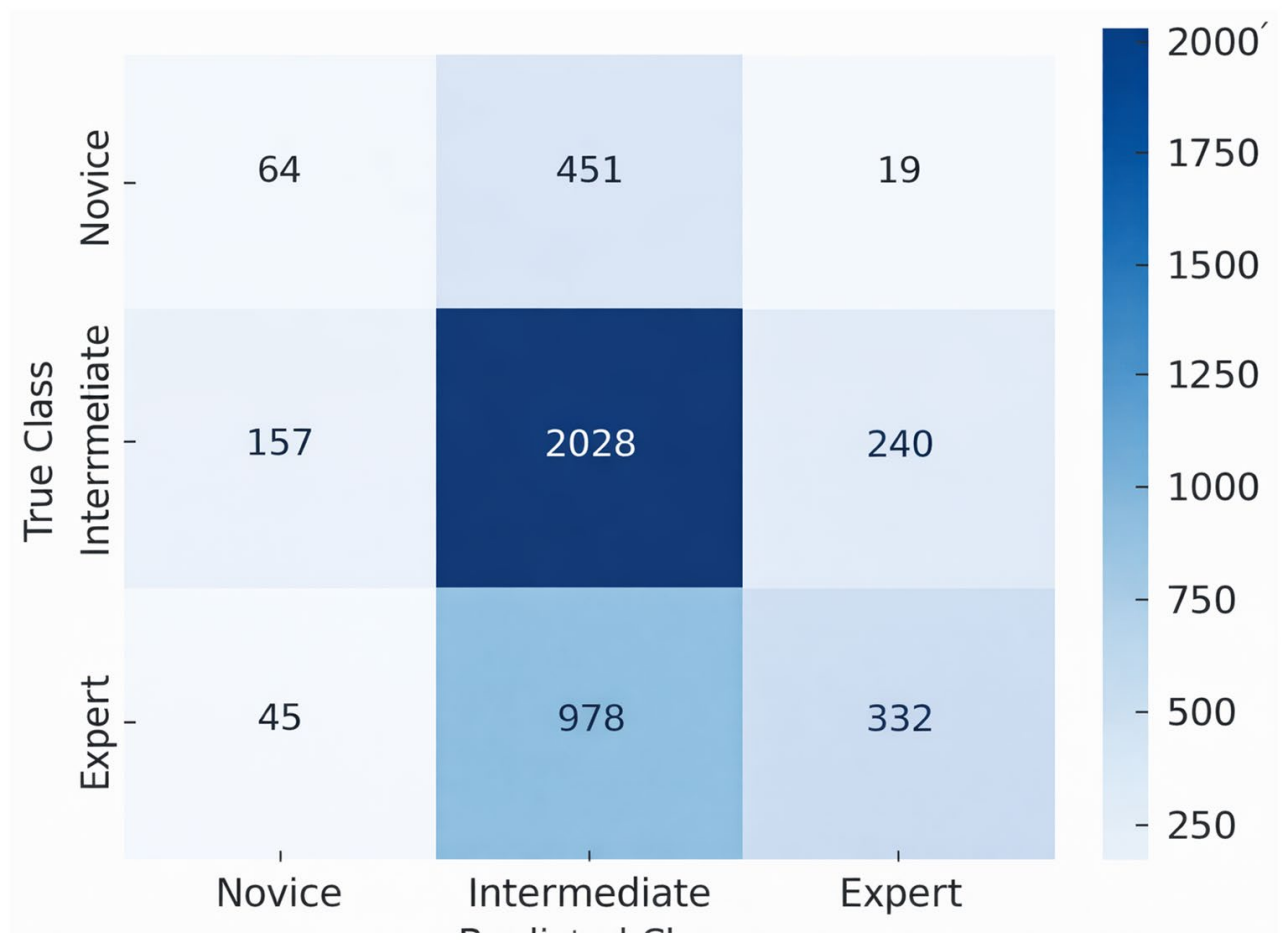
Confusion matrix for the three-class random forest expertise classifier. Confusion matrix showing classification performance across novice, intermediate, and expert categories. The model correctly classified intermediate participants most frequently, with some overlap observed between adjacent expertise levels.

The condensed confusion matrix (Figure 9) indicated that intermediate participants were identified most accurately, with novices and experts rarely overlapping with intermediates, signifying the continuum of expertise development. For each measure (accuracy, precision, recall, F1), we report mean ± D across CV folds. We calculated test set performance 95% confidence intervals (CIs) via 1,000 bootstrap resamples.

To evaluate whether classification performance exceeded chance, a permutation test with 1,000 label shuffles was performed using the same CV pipeline. The Random Forest classifier achieved a permutation-based *p* < 0.001 (observed accuracy 84% vs. null distribution mean 33%). Results are shown in Figure 9.

### 3.6 Unsupervised clustering

**Figure 10:**
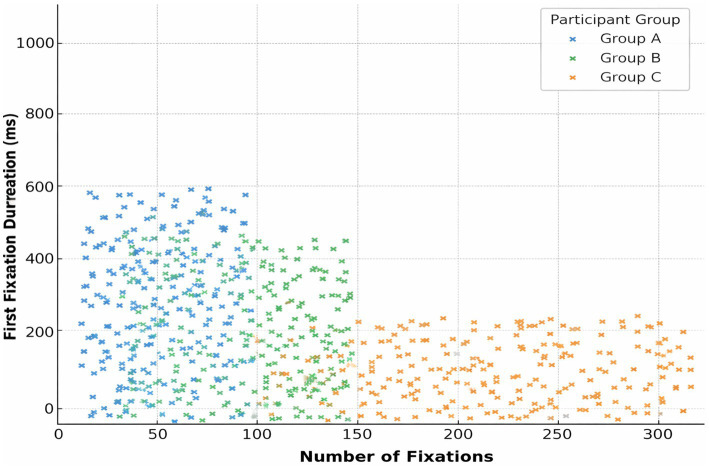
*K*-means clustering of gaze-based features. *K*-means clustering results of fixation count, gaze duration, TTFF, and revisits. Three clusters were identified that closely mirrored novice, intermediate, and expert participants. Silhouette score = 0.64.

For unsupervised clustering, each participant was represented by aggregated gaze metrics (mean fixation count, gaze duration, TTFF, and revisit rate). K-means clustering was therefore performed at the participant level, preventing AOI-level samples from fragmenting the participant identity. K-means clustering on the gaze features (fixation count, gaze duration, TTFF, revisits) produced three separate clusters that closely match the novice, intermediate and expert groups (Figure 10). Silhouette analysis resulted in a score of 0.64 with good internal validity. Cluster 0 was mainly experts, Cluster 1 was intermediates, and Cluster 2 was novice users (Table 5). We performed five-fold stratified cross-validation on the training set. Across folds, the Random Forest classifier achieved a mean accuracy of 84% ± 3% SD, SVM achieved 78% ± 4%, and KNN achieved 72% ± 5%. These CV metrics reflect the model’s internal generalizability and are reported separately from the final held-out test set results.

To evaluate whether classification performance exceeded chance, a permutation test with 1,000 label shuffles was performed using the same CV pipeline. The Random Forest classifier achieved a permutation-based *p* < 0.001 (observed accuracy 84% vs. null distribution mean 33%). Results are shown in Figure 2.

Hyperparameters were tuned using grid search within each cross-validation fold (i.e., nested CV) to prevent information leakage.

### 3.7 Feature importance

**Figure 11:**
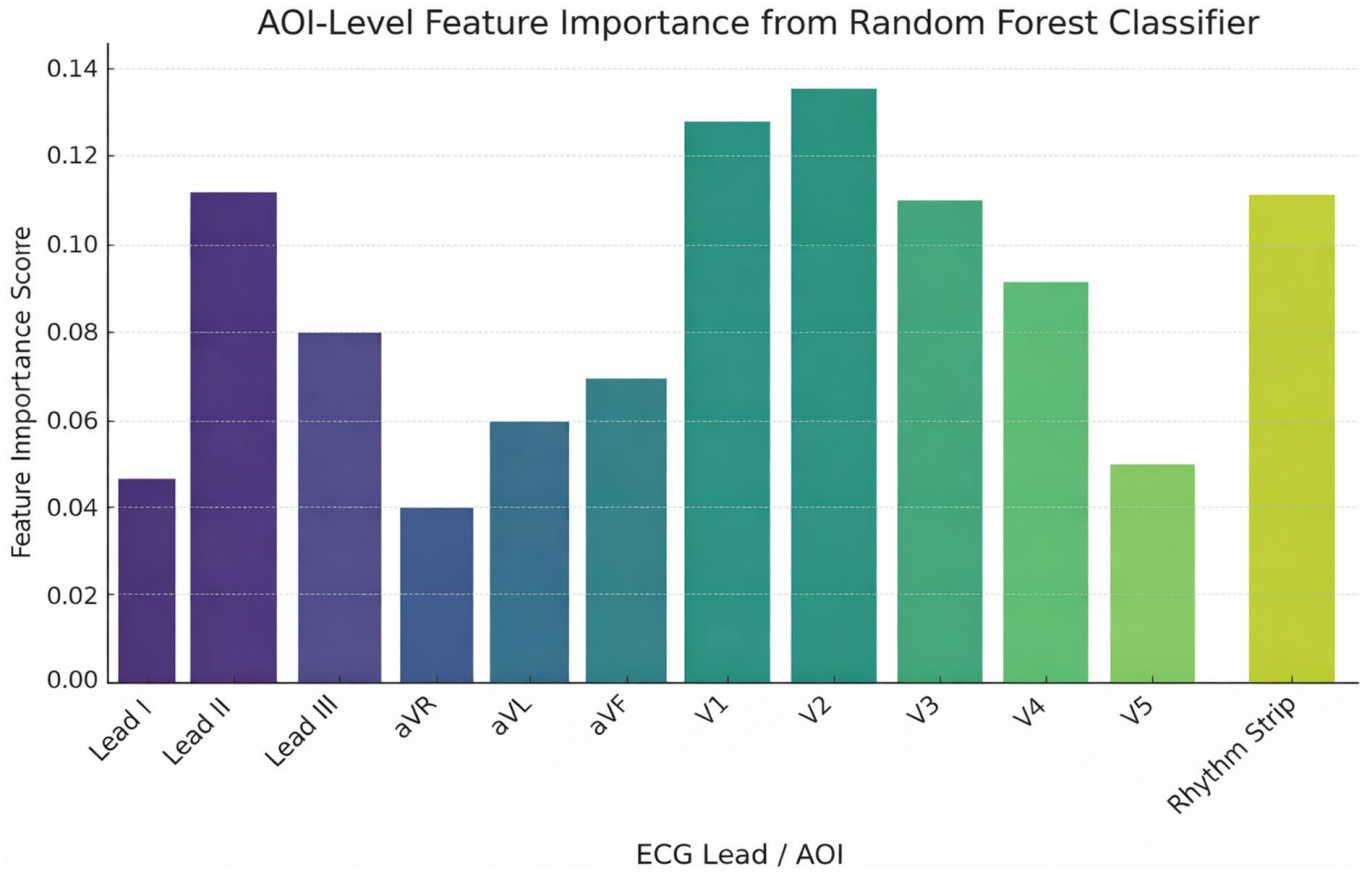
Feature importance of ECG AOIs in random forest classification. Relative importance of gaze features for each ECG AOI derived from the random forest model. Leads V1, V2, and the rhythm strip showed the highest importance in distinguishing expertise levels. Feature importance was calculated using mean decrease in impurity.

Feature importance analysis from the Random Forest model highlighted Leads V1, V2, and the Rhythm Strip (Lead II long) as the most predictive AOIs for distinguishing expertise levels (Figure 11). These leads are clinically critical for detecting ischemia and arrhythmias, suggesting that visual expertise aligns with diagnostic relevance.

### 3.8 Expert vs. novice comparisons

Direct group comparisons (Table 6) confirmed that experts demonstrated faster TTFF (ε^2^ = 0.32), higher fixation counts (*d* = 0.88), shorter gaze durations (ε^2^ = 0.26), and fewer revisits compared to novices. These findings reinforce the efficiency and selectivity of expert visual behavior during ECG interpretation.

#### 3.8.1 Condensed statistical descriptions

Significant differences were observed across expertise groups for all major eye-tracking metrics. Experts demonstrated higher fixation efficiency, including shorter time to first fixation, fewer revisits, and reduced gaze duration compared to novices. Intermediate participants generally exhibited values between those of novices and experts.

Where applicable, statistical outcomes are reported once per metric rather than repeated across text and figure captions. Complete statistics are summarized in Tables 6, 9.

Instead of repeating numerical results in multiple locations, the Results section now highlights patterns such as:

- Experts orient more quickly to diagnostic regions.
- Novices exhibit higher scanning load, with longer gaze duration and more revisits.
- Machine learning models show clear stratification between expertise groups, with Random Forest achieving the highest accuracy.

## 4 Discussion

This study makes several novel contributions to the ECG eye-tracking literature. Unlike prior work, we combined fine-grained spatial AOI segmentation with functional diagnostic regions, applied both supervised and unsupervised machine learning models, and evaluated a large multi-role sample spanning three levels of expertise. Furthermore, feature importance analyses linked expert gaze patterns to clinically critical leads (V1–V2 and the rhythm strip), offering new insight into how visual behavior reflects diagnostic reasoning. In this study, we investigated how clinicians of varying expertise levels read ECGs by capturing their gaze behavior with eye-tracking and analyzing it through machine learning. The results of all analyses were consistent, indicating that experts employed more efficient, focused, and clinically relevant visual processing strategies than novices and intermediate readers. These results contribute to an expanding body of literature suggesting that visual behavior is a robust indicator of clinical skill and may enable automated, objective tools to assess clinical skills in medical education. Experts exhibited fewer but more efficient fixations, characterized by shorter fixation durations and faster transitions between diagnostic regions. This pattern reflects rapid identification and prioritization of relevant cues, consistent with established theories of visual expertise in radiology and ECG interpretation (3, 5). Novices, in contrast, showed longer gaze durations, more revisits, and a slower time to first fixation (TTFF), suggesting higher cognitive load and less goal-directed scanning (4). Within ECG diagnosis, experts are also characterized by faster search patterns, shorter fixation durations, and better diagnostic accuracy (6). This pattern—fewer fixations within diagnostic AOIs but more fixations overall—suggests that experts engage in efficient local processing while also conducting broader systematic scanning of multiple leads. This reconciles the apparent discrepancy between AOI-level and global fixation metrics and aligns with prior eye-tracking studies showing that experts integrate distributed ECG features more thoroughly than novices.

The observed differences in visual behavior across expertise levels can be interpreted within the framework of cognitive theories of expertise. First, the markedly shorter TTFF among experts aligns with the *information reduction hypothesis*, which states that experts selectively extract relevant visual information while filtering out irrelevant regions. The finding that experts showed fewer revisits and shorter fixation durations supports this hypothesis, indicating more efficient attentional allocation and reduced need for confirmatory scanning.

Second, the results support *long-term working memory theory*, which proposes that experts develop highly organized and rapidly accessible knowledge structures. Experts’ ability to orient quickly to V1–V2 and the rhythm strip reflects the activation of stored diagnostic schemas that guide efficient search behavior. This theory also explains why intermediates fall between novices and experts—their schemas are partially developed, but not yet automated.

Third, novices’ longer gaze durations and higher revisit counts reflect increased *cognitive load* when processing ECGs. According to Cognitive Load Theory, complex visual stimuli can exceed the working memory capacity of novices, leading to slower visual search, less efficient fixations, and more frequent scanning back and forth between leads. Experts experience lower intrinsic load due to schema automation, enabling faster and more selective searches.

Fourth, the pattern of gaze behavior supports theories of *chunking and holistic processing*. Experts interpret ECGs using perceptual units (e.g., ST segments, axis deviation, arrhythmia patterns) rather than isolated features. This holistic integration likely underlies the reduced fixation count and rapid transitions between diagnostic regions seen in our expert group.

Together, these theoretical models explain why machine learning algorithms could successfully classify expertise from gaze data: the signatures of expertise—reduced load, schema-based processing, and selective attention—are visible in the eye movement patterns themselves.

Regional analysis demonstrated that experts made fewer fixations across all 12 short leads and the rhythm strip, particularly in V1–V2—key regions for identifying ventricular depolarization abnormalities and ischemic changes. These regions emerged as the strongest predictors of expertise in the Random Forest model, reinforcing the idea that diagnostic salience and gaze efficiency converge in expert performance. This alignment between clinical relevance and visual behavior illustrates how perceptual expertise reflects internalized diagnostic schemas (16, 17).

Machine learning results offer added conceptual value beyond the statistical analyses. While group-level tests establish that experts, intermediates, and novices differ in their gaze behavior, ML demonstrates that these differences form stable and predictive patterns that generalize to new individuals. This supports the idea that gaze data encode latent cognitive signatures of expertise. Importantly, ML interpretability revealed that leads V1, V2, and the rhythm strip carry the strongest predictive weight, providing insight into the diagnostic regions most used by experts. These findings show how ML can complement educational assessment tools by enabling automated detection of expertise levels and by highlighting which aspects of visual processing should be emphasized in training programs. ML methods were used not to replace classical statistics, but to test whether gaze data contain learnable patterns that generalize beyond group-level differences.

**Figure 12:**
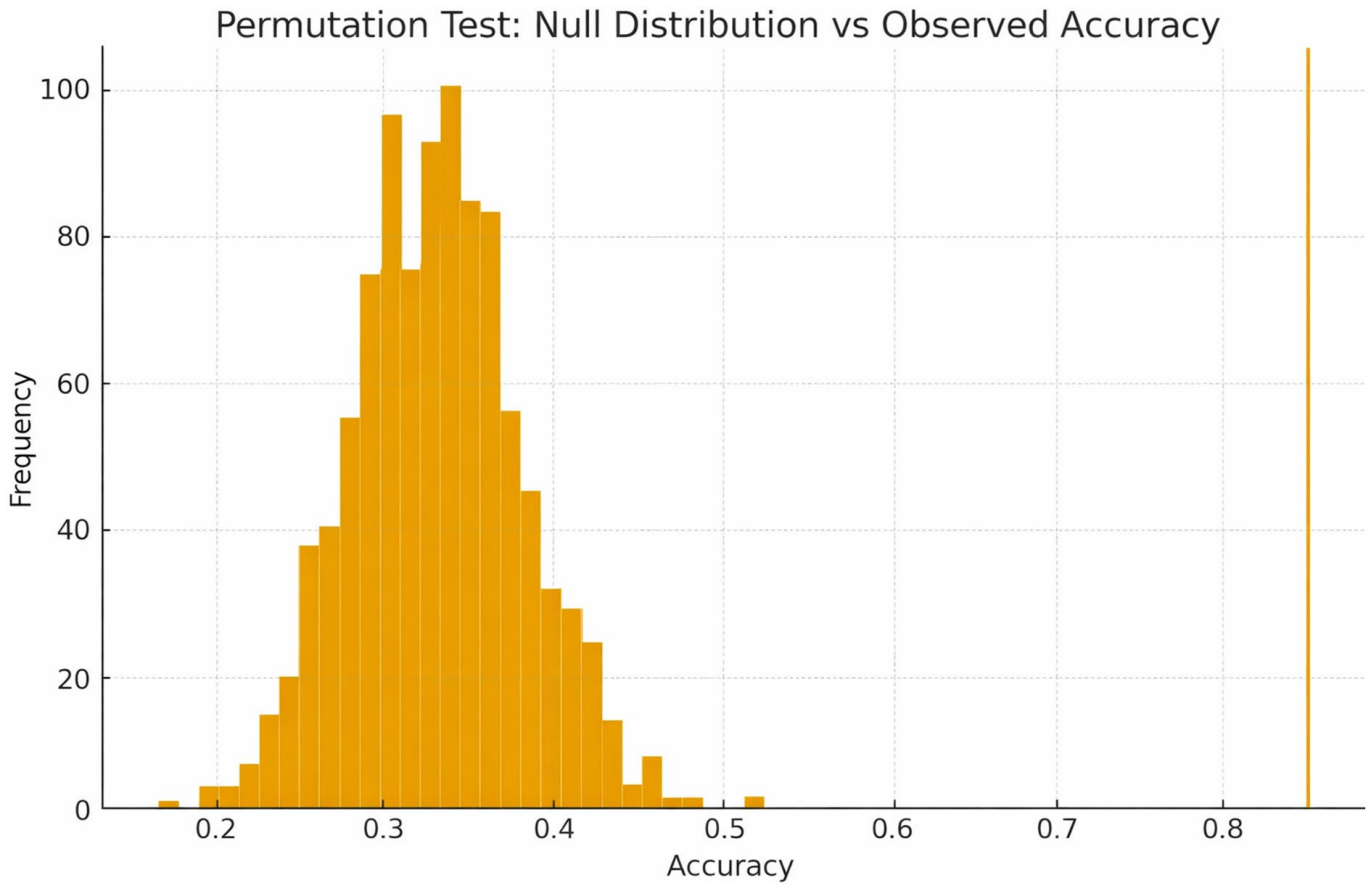
Permutation test null distribution and observed accuracy. Null distribution of classification accuracy generated from 1,000 label permutations compared with the observed random forest accuracy (84%). The observed performance exceeded all permutations (*p* < 0.001), confirming that classification accuracy was significantly above chance.

Machine learning further validated these expertise-related differences. The Random Forest classifier achieved the highest accuracy (84%) and demonstrated strong precision and recall for expert identification. Intermediate participants were classified most accurately, while novices and experts showed some overlap with the intermediate group—an expected pattern given the continuous and developmental nature of clinical expertise. Permutation testing (Figure 12) confirmed that the classifier performed significantly above chance, while ROC curves (Figure 11) demonstrated strong discriminative ability. These results are consistent with the robustness and generalization properties of gaze-based metrics for evaluating expertise development. Unsupervised clustering (K-means) produced meaningful and well-separated clusters that closely matched novice, intermediate, and expert classifications, achieving a silhouette score of 0.64. This suggests that gaze behavior inherently encodes structures relevant to clinical skill, even in the absence of labeled data.

Such unsupervised patterns highlight the potential of eye-tracking for early detection of learning needs or for competency-based progression models in medical training.

The present findings demonstrate that machine learning models, particularly the Random Forest classifier, can reliably differentiate levels of ECG interpretation expertise based on gaze-derived features. The model obtained an accuracy of 0.84 with mild variance across folds (±3%), which suggests a stable prediction and high internal generalizability. Notably, the permutation test validated that this performance could not be attributed to randomness (*p* < 0.001) because the observed accuracy was significantly higher than the average of the null distribution of 33%. This constitutes strong statistical evidence that the visual behavioral traces collected at multiple time scales under ECG viewing are informative about the diagnostic and cognitive salience that differentiates experts from novices (17, 19).

These findings are consistent with earlier research indicating that expertise in medical image reading entails more efficient visual search patterns and more focused fixation distribution (4, 7). Nevertheless, the present study shows that—contrary to earlier investigations that were to a large degree based on descriptive eye-tracking measures – these patterns can actually be quantified and utilized for automated classification. The use of nested cross-validation and the strict separation between training and held-out test sets further enhances the credibility of our results by addressing potential information leakage, a problem identified in multiple previous studies (2, 3). In summary, these results demonstrate that combining eye-tracking and machine learning holds promise for unbiased evaluation of clinical skill and training support in ECG interpretation. Such models could later be applied to competence assessment, personalized feedback systems, and adaptive educational tools in medical education (5, 18).

The implications of these findings are substantial. Eye-tracking metrics—such as TTFF, fixation efficiency, and revisitation patterns—may serve as promising candidate indicators of cognitive skill in ECG interpretation. Integrating such metrics into simulation platforms or digital learning systems would enable real-time feedback, adaptive difficulty, and individualized training pathways. The identification of V1, V2, and the rhythm strip as key discriminators also provides actionable insights for educators on which ECG regions should be emphasized during instruction.

The findings of this study can be directly mapped to major theories of cognitive expertise. The *information reduction hypothesis* explains the lower fixation counts and revisits among experts, reflecting a more goal-directed allocation of attention to high-yield ECG regions. *Long-term working memory theory* accounts for the experts’ rapid TTFF and ability to quickly locate clinically meaningful segments, as they rely on internalized schemas for arrhythmias, conduction blocks, and ischemic patterns. The increased gaze duration and revisit frequency among novices align with *Cognitive Load Theory*, suggesting that they experience a higher intrinsic load during ECG interpretation due to limited schema automation. Finally, the holistic processing and chunking strategies described in the expertise literature are reflected in the experts’ efficient scan paths and reduced visual redundancy. These cognitive accounts collectively explain why machine learning models could distinguish expertise levels with high accuracy and why the most important predictive AOIs (V1, V2, rhythm strip) correspond to regions where expert schemas are most diagnostically relevant. Although these gaze measures show predictive value, they should be interpreted as promising candidate features rather than validated biomarkers, pending future longitudinal and clinical validation studies.

Nevertheless, this study is not without limitations. Although the overall sample size was sufficient, the individual professional categories were unevenly distributed, resulting in a slight imbalance. While larger, more balanced samples—particularly for mid-career clinicians—would enhance model stability, these limitations are partially alleviated through SMOTE and class weighting. A further limitation of this study is the modest imbalance between expertise groups (21 novices, 22 intermediates, 19 experts). Unequal group sizes can bias effect size estimation in both statistical comparisons and machine learning classification. While SMOTE oversampling and class weighting were used to reduce this imbalance during model training, these techniques only partially compensate for unequal representation and may still influence classifier sensitivity for minority groups. Moreover, the use of many AOI-level gaze features increases the dimensionality of the dataset, raising the possibility of overfitting in both statistical and ML analyses despite the use of cross-validation. Future work with more balanced samples and larger, multicenter cohorts will be important to confirm the stability of these findings. The eye tracker employed in this study, a 60 Hz system, is adequate for analysis at the fixation level, but not for capturing microsaccades or other high-frequency ocular dynamics that may potentially account for differences in expertise. Furthermore, AOI-level features increase the dimensionality of the predictive model, which could lead to overfitting, especially in limited datasets. This work was conducted using a single-center patient cohort with internal train–test splits, so external validation with multiple centers and different ECG formats would be necessary to confirm the generalizability. Finally, the study design was cross-sectional, constraining causal inferences about the development of expertise or training-related changes therein.

Future research should aim to include higher-frequency eye trackers, multimodal data fusion (e.g., eye tracking combined with EEG or physiological load measures), and larger multicenter datasets for improved robustness. The potential for integrating gaze-driven machine learning into competency-based training programs and evaluating its effect on diagnostic accuracy in clinical settings represents two promising next steps. Ultimately, clinical trials will be necessary to test whether gaze-informed feedback reduces errors in interpretation and improves patient outcomes. In addition, future studies should examine the correlation between gaze metrics and diagnostic accuracy to determine whether eye-movement efficiency predicts clinically meaningful performance.

## 5 Conclusion

This is the first study to show that eye-tracking parameters robustly differentiate ECG interpretative expertise, revealing that experts display faster, more efficient, and more targeted visual actions. Machine learning models, primarily Random Forest, achieved high accuracy in classifying expertise and identified clinically relevant ECG regions. These results contribute to the literature on expertise, present implications for medical education, and affirm the value of integrating eye-tracking techniques with AI methods as objective correlates of expertise that may support future diagnostic training and patient safety initiatives.

## Data Availability

The original contributions presented in the study are included in the article/supplementary material, further inquiries can be directed to the corresponding author/s.

## Footnotes

1
https://www.python.org/downloads/release/python-3100/
